# Study on the Macro-/Micrometric Characteristics and Mechanical Properties of Clayey Sandy Dredged Fill in the Guangdong Area

**DOI:** 10.3390/ma17236018

**Published:** 2024-12-09

**Authors:** Qiunan Chen, Xiaodi Xu, Ao Zeng, Yunyang Yan, Yan Feng, Kun Long, Chenna Qi

**Affiliations:** 1 College of Civil Engineering, Hunan University of Science and Technology, Xiangtan 411201, China; xiaodixu@mail.hnust.edu.cn (X.X.); fengy8891@163.com (Y.F.); aitekn@163.com (K.L.); 18773326735@163.com (C.Q.); 2 Hunan Province Key Laboratory of Geotechnical Engineering Stability Control and Health Monitoring, Hunan University of Science and Technology, Xiangtan 411201, China; 3 Hunan Shang Shang Municipal Construction Development Co., Changsha 410011, China; 13786226133@163.com (A.Z.); 13787022112@163.com (Y.Y.)

**Keywords:** dredged fill, clayey sand, triaxial shear, triaxial creep

## Abstract

The study of dredged fill in Guangdong (GD), China, is of great significance for reclamation projects. Currently, there are relatively few studies on dredged fill in Guangdong, and there are many differences in the engineering characteristics of dredged fill foundations formed through land reclamation and natural foundations. In order to have a more comprehensive understanding of the physico-mechanical properties of blowing fill in the coastal area of GD and to understand the effect of its long-term creep row on the long-term settlement and deformation of buildings, the material properties, microstructure, elemental composition, triaxial shear properties, and triaxial creep properties of dredged fill in Guangdong were studied and analyzed through indoor geotechnical tests, scanning electron microscopy (SEM), X-ray diffraction (XRD), and conventional triaxial shear tests and triaxial creep tests. The test results showed that the Guangdong dredged fill is characterized by a high water content, high pore ratio, and high-liquid-limit clayey sand, and the mineral composition is dominated by quartz and whitmoreite. The scanning electron microscopy results showed that the particles of the dredged fill showed an agglomerated morphology, and the surface of the test soil samples had scaly fine flakes and a fragmented structure. In the triaxial shear test, the GD dredged fill showed strain hardening characteristics, and the effective stress path showed continuous loading characteristics; the consolidated undrained shear test showed that the GD dredged fill had shear expansion characteristics under low-perimeter-pressure conditions. It was found that, with an increase in bias stress, the axial strain in the consolidated undrained triaxial creep test under the same perimeter pressure conditions gradually exceeded the axial strain in the consolidated drained triaxial creep test. The results of this study are of theoretical and practical significance for further understanding the mechanical properties of silty soils in the region and for the rational selection of soil strength parameters in practical engineering design.

## 1. Introduction

With the rapid development of China’s coastal areas as a result of economic development, there is a growing demand for land resources. At present, numerous land reclamation projects are being promoted throughout China. For example, the Hengqin Reclamation Project in Zhuhai, Guangdong Province, has a reclaimed area of 31.89 square kilometers, making it one of the largest reclamation projects in China [1]. The land reclamation project in the Pudong New Area of Shanghai has been a major project since China’s reform and opening up, with a reclaimed area of 29.6 square kilometers, which has greatly boosted the city’s economic development [2]. In Nantong City, Jiangsu Province, the Haimen Reclamation Project has become the most technically difficult and largest reclamation project in the lower reaches of the Yangtze River, with its 17 square kilometers of reclaimed land and a depth of reclamation of more than 6 m [3].

Land reclamation refers to the accumulation of silted sediments and other materials on the ocean floor by means of earth blowing, which gradually forms a soil body, thus filling in the original ocean area and fixing the sediments so that they extend over land [4]. Specific methods of realizing this technique include the use of dredgers and mud pumps to blow silted sediments in the form of slurry onto the shore or within a construction site, allowing them to be gradually deposited to form foundations. Reclamation can expand the land area and transform an originally undeveloped sea area into usable land, thus expanding the land area of a city, increasing its land resources, and promoting its rapid development. Land reclamation can also extend the coastline, increase its length, facilitate the expansion of the space for marine production and utilization, and help increase the construction of ports and harbors, further promoting the development of the marine economy. It can also alleviate the problem of coastal erosion to a certain extent, prevent seawater from eroding the shoreline, and protect the shoreline’s ecological environment. Engineering geological problems such as the tilting of structures, excessive post-construction settlement of road surfaces, and instability of foundations often occur during construction work in reclaimed land areas [5,6,7,8,9,10]. Many scholars have carried out much research on these engineering geological problems; they have achieved many important results and found that the generation of the above problems is closely related to the creep characteristics of dredged fill [11,12,13,14,15]. Therefore, it is of great significance to study the creep and deformation characteristics of dredged fill.

A large number of scholars have adopted different means of carrying out research on blowing soil in different coastal regions, such as Liu Ying [16], who compared the basic properties of dredged fill in Lianyungang and Qingdao, found that there is a big difference between the two, and proposed that the difference lies in the source of blowing silt and different hydraulic conditions. Su Xiaoping [17] compared the basic properties of dredged fill in Huanghua, Caofeidian, Tianjin, and Dalian in Bohai Bay and found that there are various types of blow-fill soils in the Bohai Sea area; the mineral composition and elemental composition of blow-fill soils in the four areas were explored. Yang Aiwu [18,19,20] conducted various stress path triaxial tests and creep tests on the blow-fill soils in Binhai New Area, Tianjin, and revealed the mechanical properties of the dredged fill in this area. Lei Huayang [21] conducted creep tests on silty soil and blowing fill in Binhai New Area and determined the influence of factors such as the loading ratio and specimen height on the consolidation creep properties of dredged fill. For blow-fill calcareous sand, Ye Jianhong [22] carried out triaxial creep tests, and the test results showed that there was particle fragmentation under high peripheral pressure. Q.N. Chen [23] carried out a consolidated drained triaxial test and consolidated undrained triaxial test on soft clay soil in the Dongting Lake area and proposed a fractional-order constitutive model containing three damage factors. P.V. Lade [24] conducted triaxial creep tests on Caribbean coral sands, analyzed the patterns of axial change and volume change during creep, and found that a decrease in stress delayed creep. There are big differences between the dredged fill of new blow-fill soil and the naturally deposited soil in terms of genesis, structure, mineral composition, and spatial distribution, and there are also some differences between dredged fill and blow-fill soil in different coastal areas.

The study of dredged fill in Guangdong is necessary due to the variability in the density, liquid–plastic limit, and particle gradation of dredged fill in coastal areas. This study analyzes the material properties, elemental composition, microstructure, compression and creep properties, and triaxial shear properties of the dredged fill in the Guangdong area by means of basic physical and mechanical property tests, X-ray diffraction, scanning electron microscopy, conventional consolidated undrained and drained triaxial tests, and conventional consolidated undrained and drained triaxial creep tests, with the aim of gaining a more comprehensive understanding of the physical and mechanical properties of blowing fill in the coastal areas of Guangdong and understand the effects of its long-term creep on the long-term settlement and deformation of buildings.

## 2. Macro- and Microproperties of Dredged Fill

### 2.1. Basic Physical Properties of Dredged Fill

**Figure 1:**
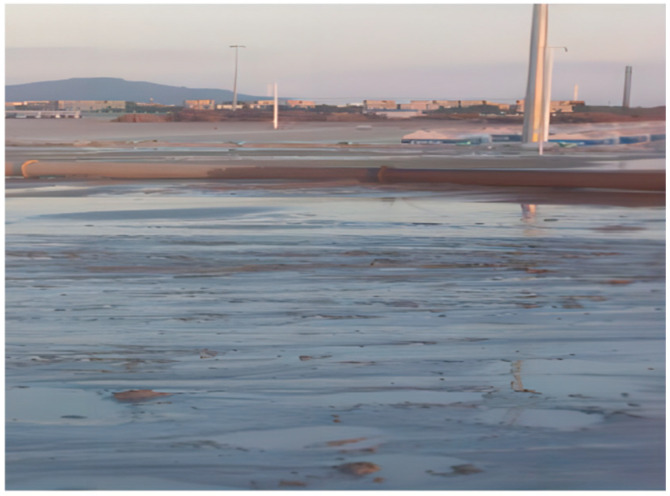
Soil extraction site.

**Figure 2:**
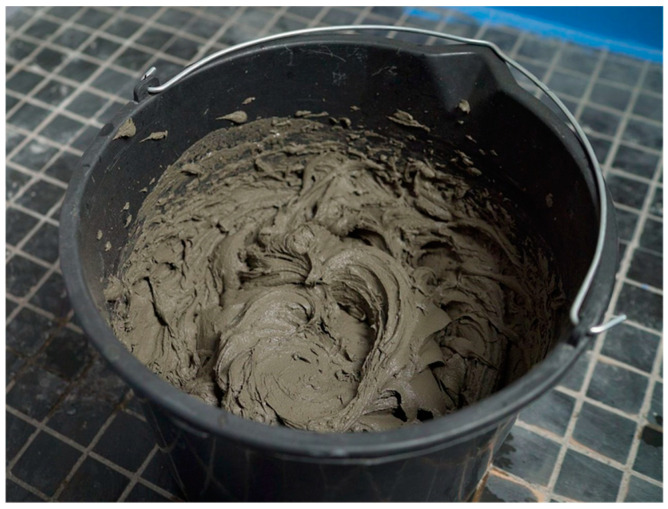
Dredged fill.

**Figure 3:**
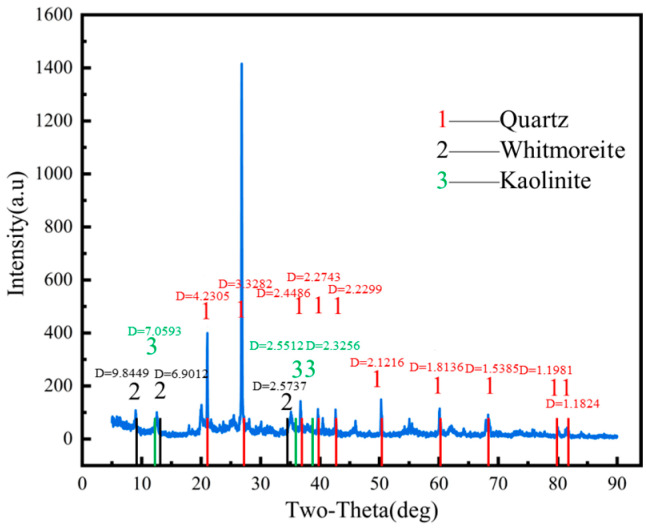
XRD pattern.

**Figure 4:**
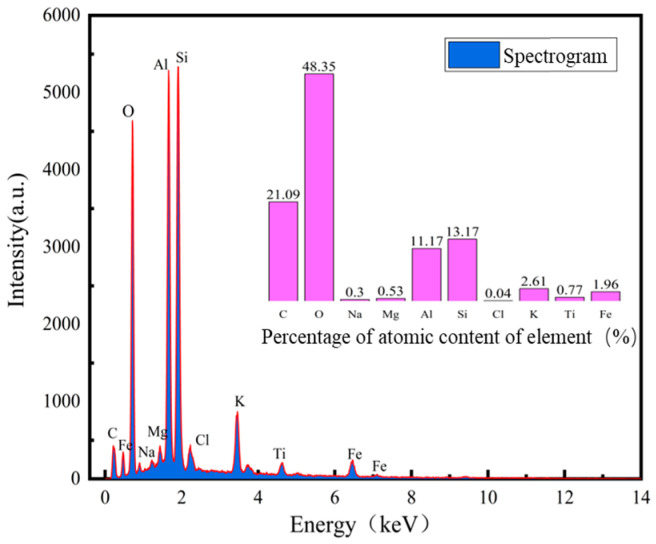
Elemental analysis of soil samples.

The soil used in this study was taken from the reclamation project of Humen Port Terminal in Dongguan City, Guangdong Province. The sampling location is shown in Figure 1, and the soil samples are shown in Figure 2; they were grayish-blackish brown, saturated, relatively homogeneous, and cohesive, with some shell fragments and humus in the soil, and the samples were air-dried to a light brownish and grayish color. The drying method was used to measure the water content of the soil, the ring knife method was used to measure the natural density of the soil, the specific gravity of the soil was obtained by the specific gravity bottle method, the dry density of the soil and the porosity ratio were calculated, the liquid limit, plastic limit, plasticity index, and liquidity index of the soil were obtained by the liquid–plasticity limit tester method, and the particle grading was obtained by the sieve method and the liquid pipette method. The basic physical property indexes of the soil samples obtained from the experiment are shown in Table 1, and the water content and grain size composition of the soil boundary are shown in Table 2, which shows that the dredged fill was a high-liquid-limit clayey sand with high water content and high compression, and the main constituent of the dredged fill was sand; the content of the clay grains in the fine-grained group was higher than that of pulverized grains. The XRD test instrument was a Bruker X-ray diffractometer (D8 ADVANCE from Rheinstetten, Germany), and the analysis software was the JADA software Release 6.5.26. The wavelength was 1.54059, and the angle of incidence was 0 to 90°. The XRD results of the mineral composition of the dredged fill in this study are shown in Figure 3, and the soil samples were mainly composed of non-clay minerals (quartz, whitmoreite), which accounted for about 85% of the content. The energy-dispersive spectrometer used was a TESCAN MIRA LMS scanning electron microscope from Brno, Czech Republic. The results of the EDS of the surface elemental composition of the dredged fill in this study are shown in Figure 4, with oxygen (O), silicon (Si), aluminum (AI), and carbon (C) accounting for the largest proportion of the sample, indicating that they were the main components of the clayey sandy dredged fill.

In Table 2, it can be seen that blow-fill soils from different regions have different liquid–plastic limits and particle gradations. The gradation of the sand particles in the blow-fill soil in Dongguan, Guangdong Province, is obviously higher than that of many kinds of blow-fill soils in the literature [17].

### 2.2. Microstructure of Dredged Fill

**Figure 5:**
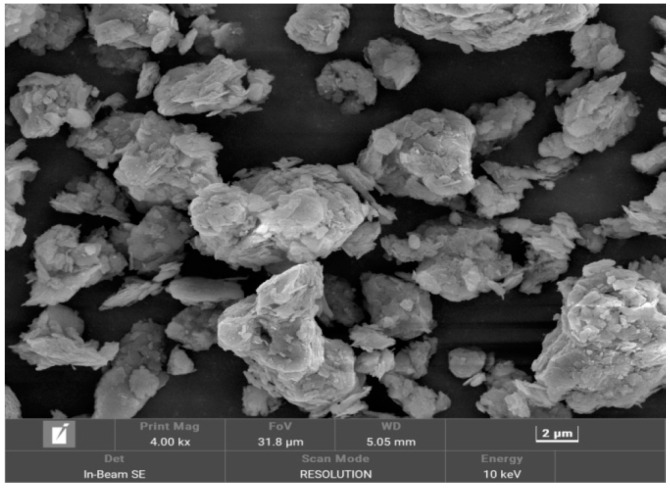
Soil samples magnified 4000 times.

**Figure 6:**
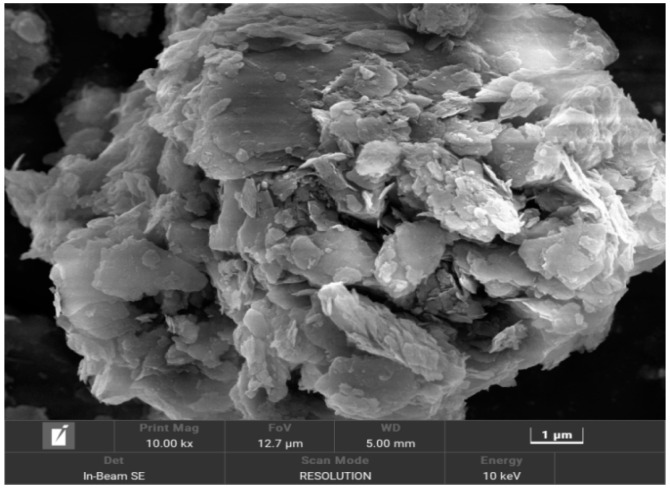
Soil samples magnified 10,000 times.

In this test, the dry powder of dredged fill was scanned using a Czech TESCAN MIRA LMS scanning electron microscope, and the scanning results are shown in Figure 5 and Figure 6. The samples were magnified by up to 4000 and 10,000 times in the scanning electron microscope test. As can be seen in the figures, the soil particles of the samples showed an agglomerated morphology, and the surface of the test soil samples had scaly fine flakes and fragmented structures; the basic unit of the body of these structures consisted of clay grains dominated by fragmented bodies, which were mostly arranged with each other in a side-by-side manner to form better pore connectivity. Based on a mineralogical compositional analysis, this structure may be a clastic deposit formed after the weathering of whitmoreite.

## 3. Pilot Program

### 3.1. Triaxial Compression Test

**Figure 7:**
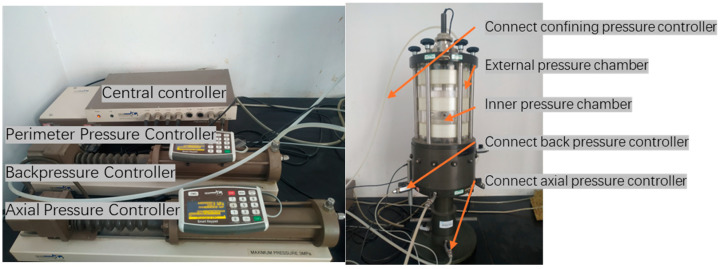
GDS triaxial stress path test.

The test was carried out using the UNSAT unsaturated triaxial soil meter manufactured by GDS, Hampshire, UK, which is shown in Figure 7. It has a pressure range of 2 MPa, volume range of 200 cc, pressure measurement resolution of 0.2 kPa, volume measurement resolution of 1 mm^3^, measurement accuracy for pressure of ≤0.1% of the full scale and for volume of ≤0.25% of the full scale, ambient temperature range of 10 °C to 30 °C, maximum control filling/draining speed of up to 500 mm^3^/s, and fast filling/draining speed of up to 1200 mm^3^/s.

The triaxial shear test was divided into a consolidated drained (CD) triaxial shear test and a consolidated undrained (CU) triaxial shear test. Remolded soils were used because the dredged fill itself did not have a natural structure. The samples were loaded as much as possible into the test apparatus using the original particle gradation and dried at 105 °C for at least 8 h before use. Considering that the depth of dredged fill is generally 6–8 m, the peripheral pressure during the triaxial test was set to 50–150 kPa. During the test, saturator saturation and counterpressure saturation were carried out first, isotropic consolidation was carried out under a certain peripheral pressure *σ*_3_, and the completion of consolidation was judged on the basis of a pore pressure dissipation of at least 95%. The perimeter pressure was kept constant after the consolidation was completed; the drain valve was closed/opened for undrained/drained shear, respectively; the axial displacement was zeroed before the shear started; and the shear rate was 0.00987%/min in the CU test and the CD test. The test process was set up with reference to the ‘Standard for Geotechnical Test Methods: GB/T 50123-2019’ [25].

### 3.2. Triaxial Creep Test

**Figure 8:**
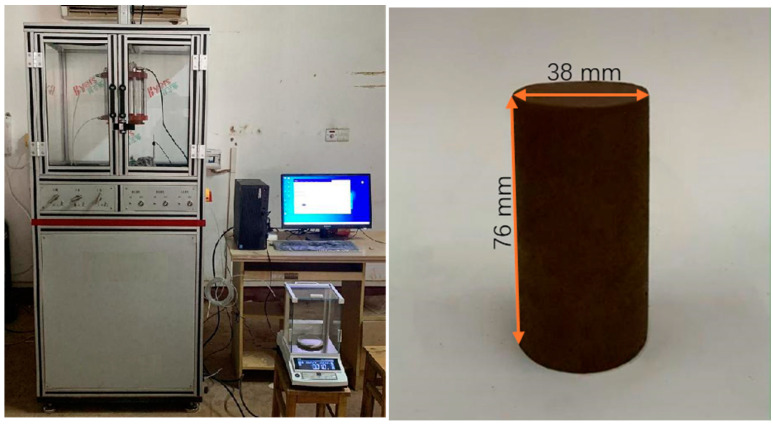
Triaxial creep test instrument and a tested soil sample.

The loading equipment used in the experiment was a TSW-30-type soil creep (rheology) triaxial tester produced by Changchun Chaoyang Testing Instrument Co., Ltd. (Changchun, China), with a maximum axial pressure of 30 kN and a maximum restraining pressure of 2 Mpa. The amount of deformation could be monitored as follows: 0–20 mm in the axial direction, with a measurement accuracy of ±0.5% and a deformation speed control range of 0.01–5 mm/min. The tester was mainly used to test the rheological characteristics of the soil under the action of triaxial stress, as well as the creep characteristics under triaxial stress. It could be controlled with a constant load and constant deformation under constant peripheral pressure, and it could test the change in the force or deformation of the specimen under the above triaxial loading conditions, as well as the internal pore pressure of the specimen and the flow of pore water. The testing machine was equipped with a computer system that could automatically control the test process and collect and process the test data. The testing machine could work continuously for more than 500 h. The test apparatus and specimens are shown in Figure 8.

The triaxial creep test was divided into a consolidated drained (CD) triaxial creep test and a consolidated undrained (CU) triaxial creep test. The test was carried out in a graded loading mode, and the perimeter pressure *σ*_3_ control levels were determined to be 100, 200, 300, and 400 kPa, with a bias stress increment of 50 kPa for each level in the case of low perimeter pressure and 100 kPa for each level in the case of high perimeter pressure; the specific test parameters are shown in Table 3. CU triaxial creep tests and CD triaxial tests were carried out under different peripheral pressures. The triaxial creep stability criterion was that the deformation rate was less than 0.05 mm/day to enter the next stress level.

## 4. Analysis of the Test Results

### 4.1. Triaxial Compression Test Results

**Figure 9:**
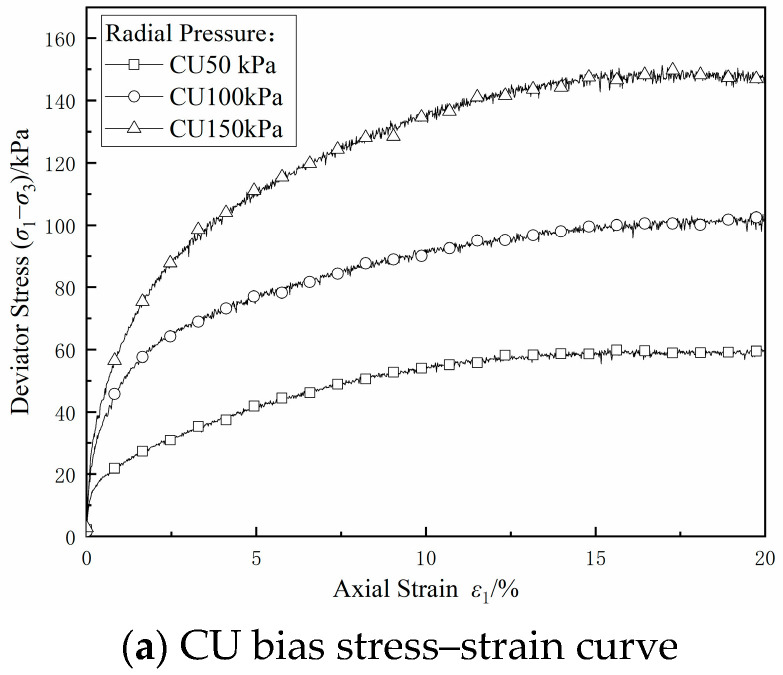

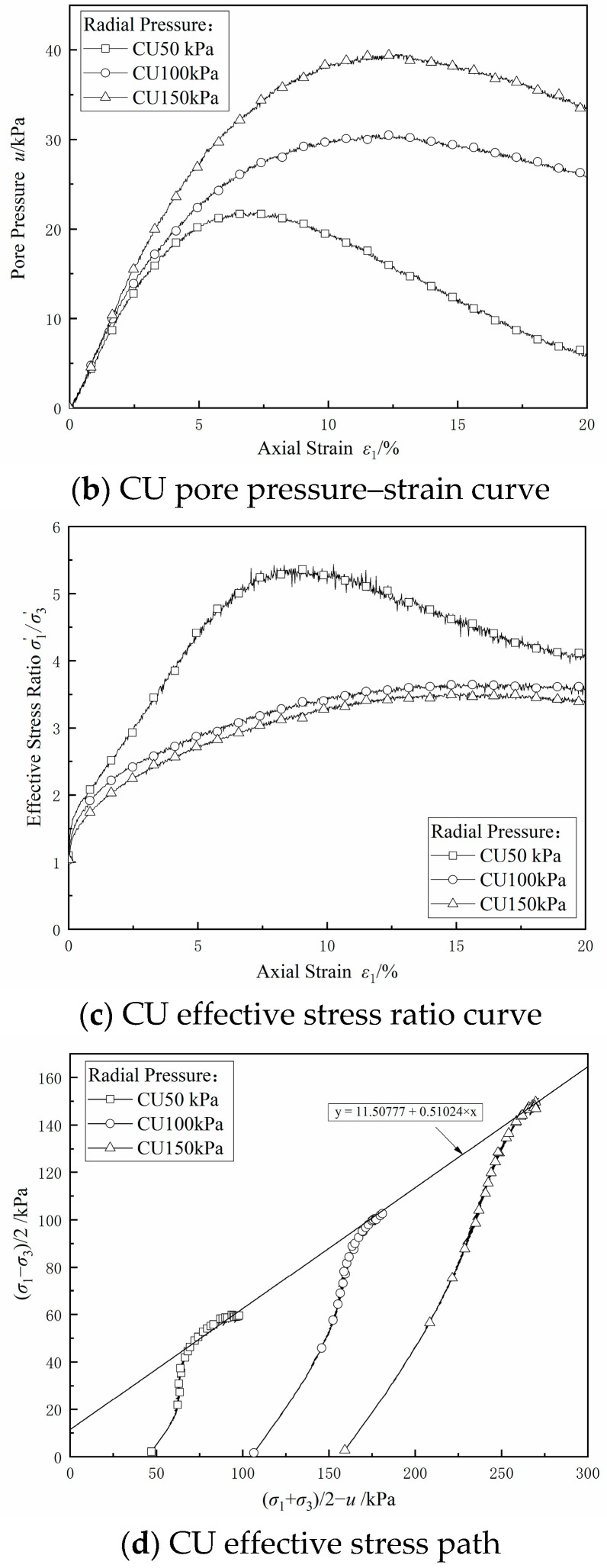
CU shear test results.

The curves of the bias stress *σ*_1_–*σ*_3_, pore pressure *u*, and effective stress ratio versus axial strain *ε*_1_ in the CU triaxial test are shown in Figure 9a–c. Under different circumferential pressures, with an increase in *ε*_1_, the bias stress firstly increased and then stabilized without an obvious peak value, and it roughly showed the characteristics of strain hardening. The pore pressure first increased and then decreased, which indicated that there was a tendency toward the shear expansion of the clayey sandy soil; under conditions of high circumferential pressure, the effective stress ratio first increased and then stabilized without an obvious peak value, and under conditions of low circumferential pressure, the effective stress ratio first increased and then decreased, thus embodying the characteristics of obvious shear expansion. Taking the average effective stress (*σ*_1_ + *σ*_3_)/2 − *u* as the horizontal coordinate and (*σ*_1_ − *σ*_3_)/2 as the vertical coordinate, the effective stress path was plotted, as shown in Figure 9d. In the shear stage, the average effective stress increased, the soil sample shows shear expansion characteristics, and the effective stress path showed loading characteristics. According to the slope, it was calculated that the inclination angle of the line connecting the damage points on the stress path diagram *α* was 30.68°, and the intercept of the damage points on the stress path diagram on the longitudinal axis *d* was 11.51 kPa. According to ‘Highway Geotechnical Test Specification: JTG 3430-2020’ [26], the effective internal friction angle *φ*′ was 36.39°, and the effective cohesion *c*′ was 14.30 kPa according to the following formula.

Formula for calculating the effective angle of internal friction:

$$\varphi \prime =\sin^{-1}\tan\alpha$$

Effective cohesion formula:

$$c\prime =\frac{d}{\cos\varphi \prime }$$

where *φ*′ is the effective angle of internal friction, *α* is the inclination of the line connecting the damage points on the stress path diagram, *c*′ is the effective cohesion, and *d* is the intercept of the damage points on the stress path diagram that are continuous on the longitudinal coordinate axis.

**Figure 10:**
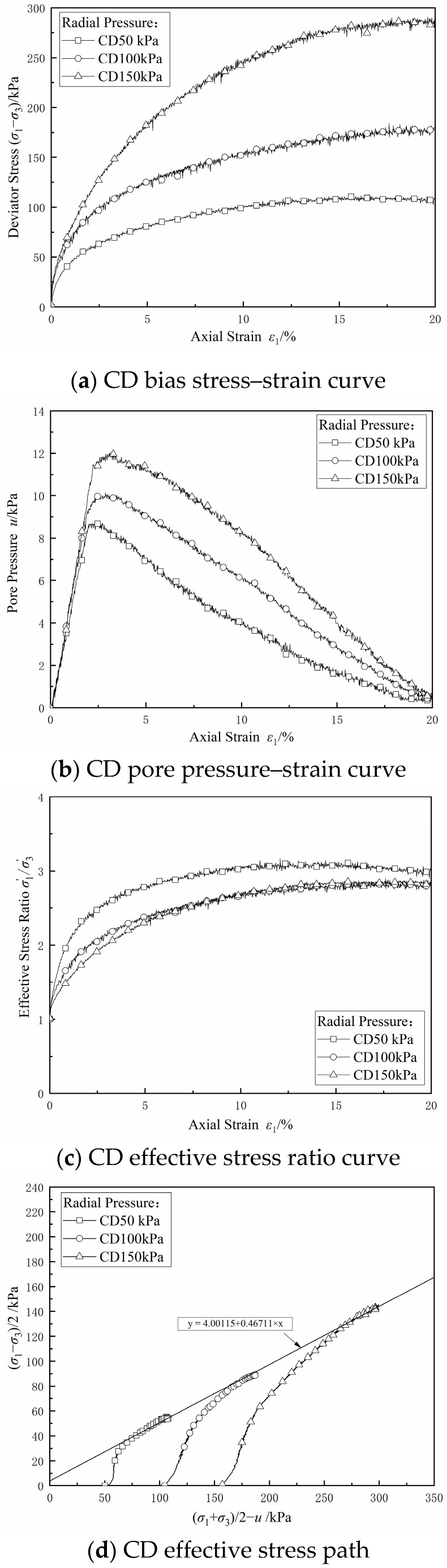
CD shear test results.

Figure 10a–c show the bias stress–strain curves, pore pressure–strain curves, and effective stress ratio–strain curves under different circumferential pressures in the CD test, respectively. Unlike in the CU test, the soil was allowed to be drained in the CD test with the increase in strain, and the pore pressure was partially dissipated, resulting in an increase in the effective stress, so the bias stress continuously increased with the increase in strain, and the strain hardening feature was more significant. The pore pressure could not be completely dissipated during shear because of the poor permeability and insufficient drainage of this dredged fill. Figure 10d shows the effective stress path in the CD test; it can be seen that the effective stress path showed loading characteristics, the inclination angle of the line connecting the damage points on the stress path diagram calculated based on the slope *α* was 27.85°, and the intercept *d* of the damage points on the longitudinal axis on the stress path diagram was 4.00 kPa, so the effective internal friction angle calculated was 31.89°, and the effective cohesive force *c* was 4.71 kPa.

**Figure 11:**
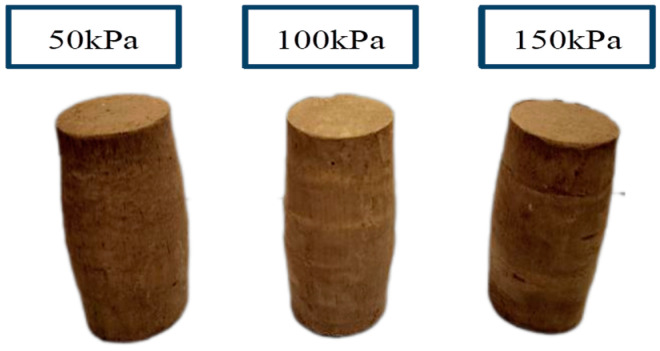
Shear end CU test specimens.

**Figure 12:**
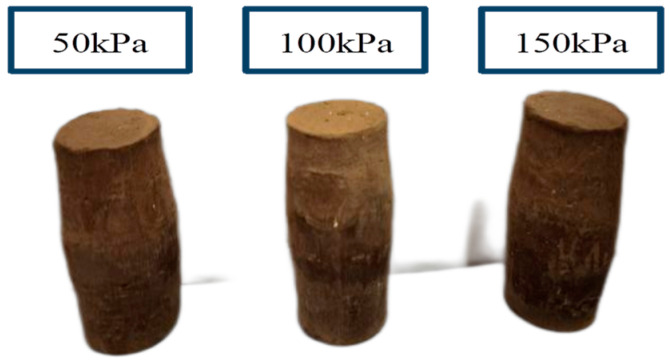
Shear end CD test specimens.

In Figure 11 and Figure 12, we can clearly observe that none of the specimens’ morphology showed obvious rupture phenomena, as they showed a drum-like morphology with a narrower top and bottom and wider center. This morphology indicated that some deformation and stress concentration occurred in the process of vertical load application.

Compared with the consolidated undrained test in [20], the maximum bias stresses of the blow-fill soil in the Dongguan area of Guangdong under the three perimeter pressure conditions were more similar to those in a triaxial test of the in situ soil of the blow-fill soil in the Binhai New Area of Tianjin, and they were greater than the results of the triaxial test of the CU of the remodeled soil.

### 4.2. Analysis of Triaxial Creep Test Results

**Figure 13:**
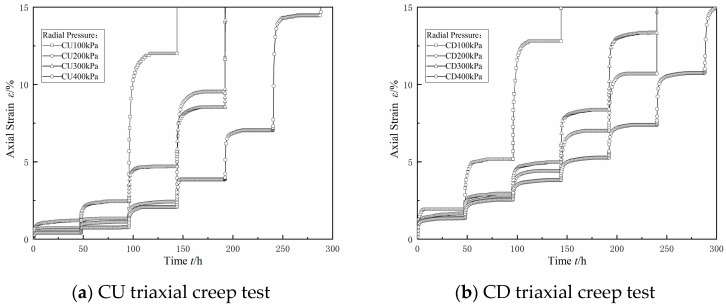
Full CU and CD triaxial creep results.

The curve of the relationship between the axial strain *ε* and time *t* in the triaxial creep test is shown in Figure 13. In the figure, it can be seen that, under the action of bias stress at all levels, the dredged fill in both the CU creep test and CD creep test showed attenuation of the creep characteristics, and the creep curve had obvious stages. Stage 1 was the transient elastic–plastic deformation stage; that is, the deformation generated by the load was applied instantly; in this stage, the soil deformation rate was large, and the deformation mainly occurred due to the transient deformation in the soil skeleton in a relatively short period of time. Stage 2 was the attenuation of the creep deformation stage; this stage had a slow increase in the strain, the deformation rate was gradually reduced, and the deformation was mainly due to the soil particles of the super-hydrostatic pore water pressure. Stage 3 was the stable creep deformation stage; at this time, the strain rate decreased to nearly 0, and the deformation tended to a stable value; the deformation was mainly due to the adjustment of the position between the soil particles.

**Figure 14:**
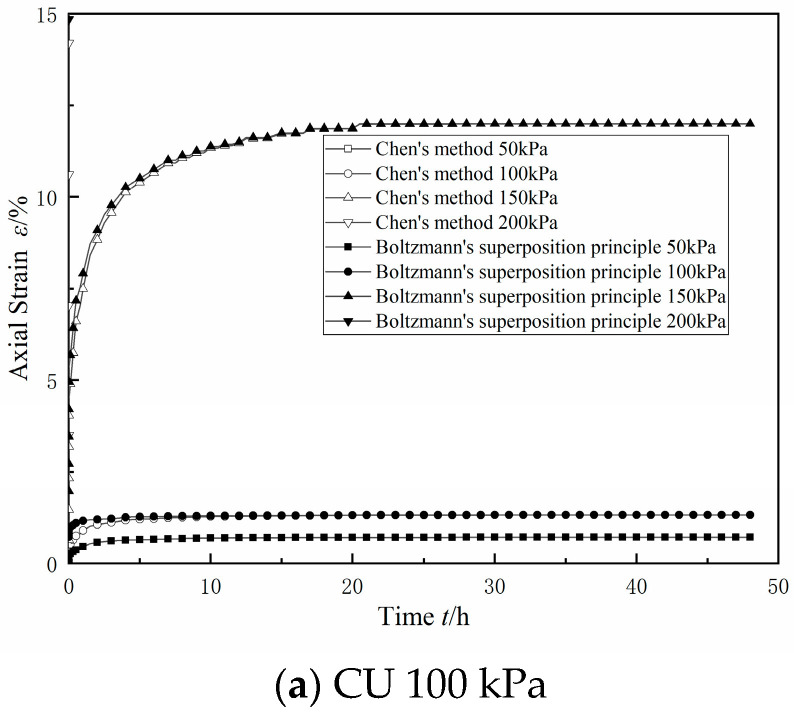

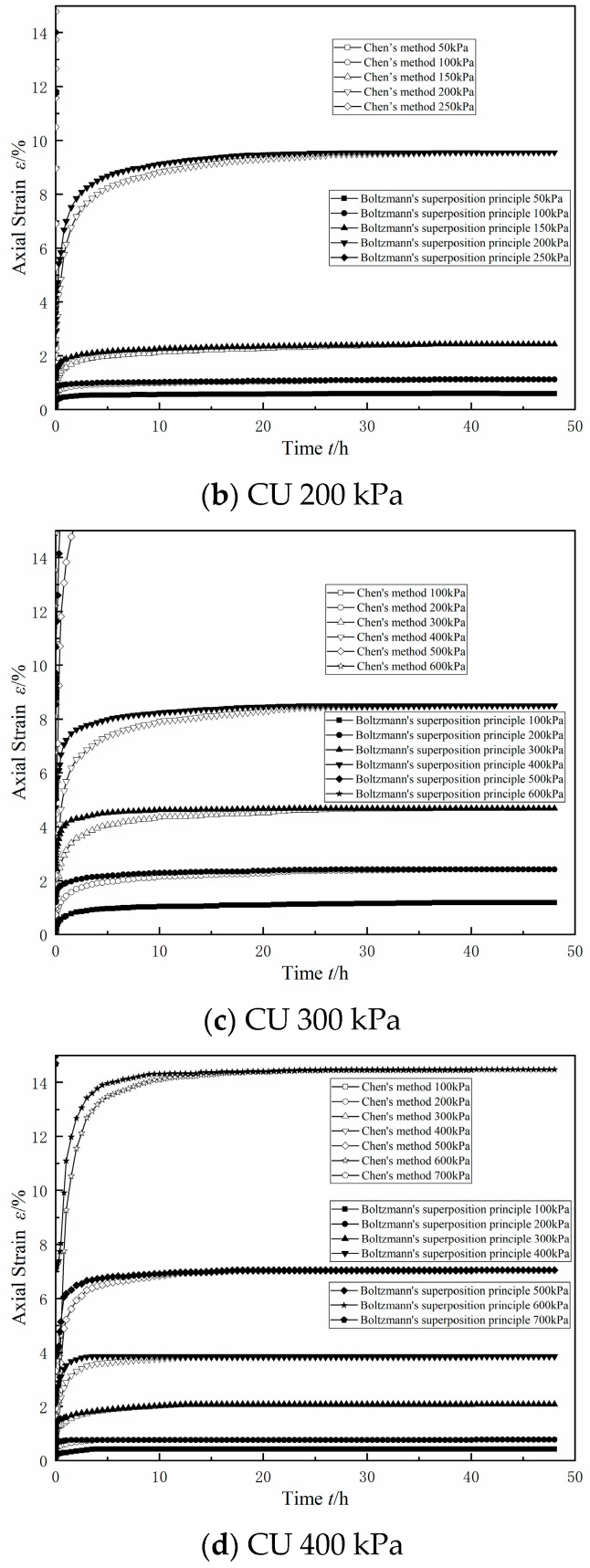
CU triaxial graded-loading creep curve.

**Figure 15:**
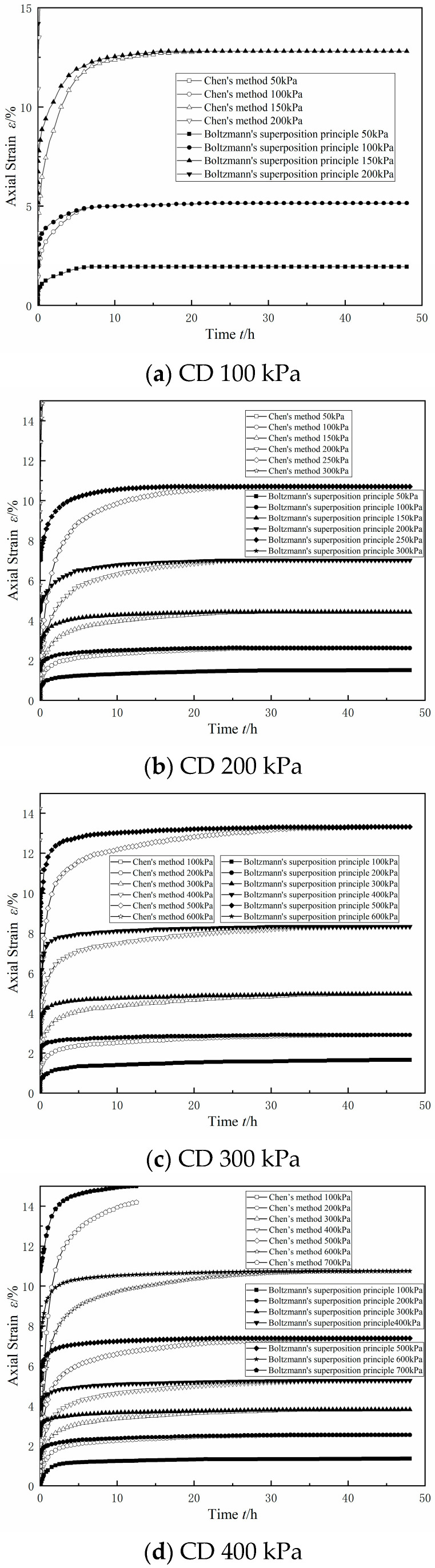
CD triaxial graded-loading creep curve.

The Boltzmann linear superposition method and Chen’s method were used at the same time to deal with the graded loading creep curve, as shown in Figure 14 and Figure 15. In the figure, for the consolidated drainage test or consolidated undrained test results when using the Boltzmann linear superposition method and Chen’s method of data processing, it can be seen that, at the first level, the bias stress conditions under the strain value were equal. With the increase in bias stress and the two methods of data processing, the difference between the two methods was also gradually increased, mainly when using Chen’s method. The difference between the two methods increased with the increase in the bias stress, which was mainly manifested in the fact that the data values processed using Chen’s method were smaller than those processed using the linear superposition method in the transient elastic–plastic deformation stage and the attenuation creep deformation stage, and the difference between the two methods was larger with the increase in the bias stress. This was consistent with the difference between Chen’s method and the Boltzmann linear superposition method found in the literature [27].

Upon comparing the results of the CU test and the CD test under the same circumferential pressure conditions in Figure 14 and Figure 15, it can be seen that the difference between the Boltzmann linear superposition method and Chen’s method for the CD test was larger than that for the CU test. In the case of low bias stress, the CU test had lower values of axial displacement than the CD test in the stable creep deformation stage. With an increase in bias stress, the axial displacement of the stable creep deformation stage in the CD test gradually exceeded that in the CU test.

## 5. Conclusions

In this study, the basic physical and chemical properties, microstructure, triaxial creep characterization, and triaxial shear properties of clayey sandy dredged fill in Guangdong, China, were systematically investigated through indoor tests. The following four conclusions were obtained.

1. There are reclamation projects in the Guangdong area of China, where the dredged fill is characterized by high water content, low density, and high saturation, and it is in a state of fluid plasticity, with a high content of sand and clay.
2. The dry powder of the dredged fill in this study showed an agglomerated morphology, and the surface of the test soil samples had scaly fine flakes and crumbly structures, which were mainly composed of quartz and whitmoreite.
3. In the triaxial shear test, the dredged fill generally showed strain hardening characteristics, with a linear relationship between the peak bias stress and the peripheral pressure, and the stress path was characterized by continuous loading. In the CU triaxial shear test, it was found that the dredged fill showed shear expansion characteristics under the condition of low perimeter pressure.
4. In the triaxial creep test, the dredged fill had an instantaneous elastic–plastic deformation stage, an attenuated creep deformation stage, and a stable creep deformation stage. Comparing the results of the CU triaxial creep test and CD triaxial creep test, it was found that the axial displacement in the stable creep deformation stage of the CU test was smaller under the condition of low bias stress, but the axial displacement in the stable creep deformation stage of the CU test always exceeded the axial displacement in the stable creep deformation stage of the CD test with the increase in bias stress.

The creep law of the dredged fill in the Guangdong coastal region, the law of the deformation of the bank slope, and the creep deformation of the terminal structure in this study are of great significance and reference value for this and other coastal regions with soft clay creep characteristics.

## Figures and Tables

**Table 1:** Basic physical and mechanical properties of soil.

Water Content/%	Natural Density/(g·cm^−3^)	Specific Gravity	Void Ratio	Dry Density /(g·cm^−3^)
78.16	1.84	2.71	1.62	1.03

**Table 2:** Soil Atterberg limit and grain size composition.

Literature	Sampling Location	Liquid Limit *w*_L_%	Plastic Limit *w*_P_/%	Plasticity Index *I*_P_	Liquidity Index *I*_L_	Soil Classification	Sand Particles		Silt Particles	Clay Particles
							2~0.25 mm	0.25~0.075 mm	0.075~0.005 mm	<0.005 mm
Herein	Dongguan, Guangdong	57.75	29.87	27.8	1.21	SHC	25.76	31.87	17.78	24.59
[17]	Huanghua	29.70	-	-	-	-	39.82		60.13	0.05
	Caofeidian	35.21	22.10	13.10	-	CL	2.31		76.77	20.92
	Tianjin	43.50	23.80	20.10	-	CL	1.80		93.71	4.49
	Dalian	51.80	30.90	20.90	-	MH	2.32		67.26	30.42

**Table 3:** Triaxial creep test program.

Test Number	Point *σ*_1_/kPa	*σ*_2_/kPa	*σ*_3_/kPa	Increment in Bias Stress per Level Δ*q_s_*/kPa	Drainage Condition
CU01	100	100	100	50	Undrained
CU02	200	200	200	50	Undrained
CU03	300	300	300	100	Undrained
CU04	400	400	400	100	Undrained
CD01	100	100	100	50	Drainage
CD02	200	200	200	50	Drainage
CD03	300	300	300	100	Drainage
CD04	400	400	400	100	Drainage

## Data Availability

The datasets presented in this article are not readily available because due to technical limitations. Requests to access the datasets should be directed to Xiaodi Xu.
